# Gateless and Capacitorless Germanium Biristor with a Vertical Pillar Structure

**DOI:** 10.3390/mi12080899

**Published:** 2021-07-29

**Authors:** Hagyoul Bae, Geon-Beom Lee, Jae Hur, Jun-Young Park, Da-Jin Kim, Myung-Su Kim, Yang-Kyu Choi

**Affiliations:** 1 School of Electrical and Computer Engineering, Purdue University, West Lafayette, IN 47907, USA; hagyoulbae@gmail.com; 2 School of Electrical Engineering, KAIST, 291 Daehak-ro, Yuseong-gu, Daejeon 34141, Korea; rjsqja1@kaist.ac.kr (G.-B.L.); dajin1202@kaist.ac.kr (D.-J.K.); mskim9993@kaist.ac.kr (M.-S.K.); 3 School of Electrical and Computer Engineering, Georgia Institute of Technology, Atlanta, GA 30332, USA; jhur45@gatech.edu; 4 School of Electronics Engineering, Chungbuk National University, Chungdae-ro 1, Cheongju, Chungbuk 28644, Korea; junyoung@cbnu.ac.kr

**Keywords:** Ge biristor, vertical memory, amorphous carbon layer, gateless structure, capacitorless structure, DRAM

## Abstract

For the first time, a novel germanium (Ge) bi-stable resistor (biristor) with a vertical pillar structure was implemented on a bulk substrate. The basic structure of the Ge pillar-typed biristor is a p-n-p bipolar junction transistor (BJT) with an open base (floating), which is equivalent to a gateless p-channel metal oxide semiconductor field-effect transistor (MOSFET). In the pillar formation, we adopted an amorphous carbon layer to protect the Ge surface from both physical and chemical damage by subsequent processes. A hysteric current-voltage (*I*-*V*) characteristic, which results in a sustainable binary state, i.e., high current and low current at the same voltage, can be utilized for a memory device. A lower operating voltage with high current was achieved, compared to a Si biristor, due to the low energy bandgap of pure Ge.

## 1. Introduction

As memory devices continue to be scaled down for high density integration, the conventional 1-transistor and 1-capacitor dynamic random-access memory (1T/1C DRAM) cell used for large storage capacity is facing process challenges. As the cell size shrinks, the aspect ratio of the cell capacitor enormously increases and the junction leakage current deteriorates [1,2]. Furthermore, reliability issues induced by off-state stress and bias-temperature instability (BTI) impede cell functionality such as on-state current (*I*_ON_) and off-state leakage (*I*_OFF_) [3]. In order to solve these technological limitations, the floating body-based dynamic random-access memory (DRAM) cell with a capacitorless structure has been under active research and development to improve fabrication simplicity and cell area scalability [4,5,6]. Such DRAM has at least three terminals: gate, source, and drain. For further aggressive scaling with a more simplified structure, a bi-stable resistor (biristor) composed of two terminals: source (emitter) and drain (collector) was reported for a gateless volatile memory device. From a structural point of view, such a biristor is categorized into two groups. One is a planar structure that was implemented on a silicon-on-insulator (SOI) wafer [7,8] for a floating body and the other is a vertical structure that was fabricated on a bulk-Si wafer [9,10]. In the vertical biristor, a p-type floating body located at the middle of the pillar was inherently made by n-type junctions positioned at a top and bottom of a pillar. Herein, the top electrode is a collector (drain), the bottom electrode is an emitter (source), and the middle floating body is a base (channel). On the other hand, germanium (Ge) based transistors have been intensively investigated in efforts to improve their electrical performance, because Ge provides both a lower energy bandgap and smaller effective mass compared with silicon (Si) [11,12,13,14,15].

One of the major goals of the Si-based biristor is the reduction of latch-up voltage (*V*_LU_) for low-voltage operation. It is difficult to reduce the driving voltage because the open-based structure of a biristor requires high voltage to trigger a high impact ionization (I.I) rate for the generation of excessive carriers. To enable low-voltage operation, an alternative material with a low energy bandgap is indispensable to increase the I.I rate. One of the candidates for the low energy bandgap material is Ge. Recently, a lower operating voltage for the I-MOS was achieved using Ge [16] and a bandgap-engineered SiGe biristor for low-voltage operation was investigated through numerical simulation [12]. Based on such results, the development of a Ge-based DRAM can be expected to achieve a lower *V*_LU_.

In this work, a capacitorless and gateless Ge-based DRAM with a vertical pillar structure comprised of p^+^ (top)-n (middle)-p^+^ (bottom) was demonstrated. In order to make the heavily doped junctions, a p+ emitter and collector were used because of high boron solubility in Ge. By employing pure Ge, a lower operating voltage and higher on-state current were achieved, compared with a Si-based biristor. The non-uniform doping profile along the vertical direction of the Ge pillar allows the unidirectional operation of the two-terminal biristor in a cross-bar array, also resulting in the blocking of current leakage via the sneaky path. Furthermore, the pillar-shaped vertical structure can be an optimized structure to minimize the area of the biristor.

## 2. Device Fabrication

**Figure 1:**
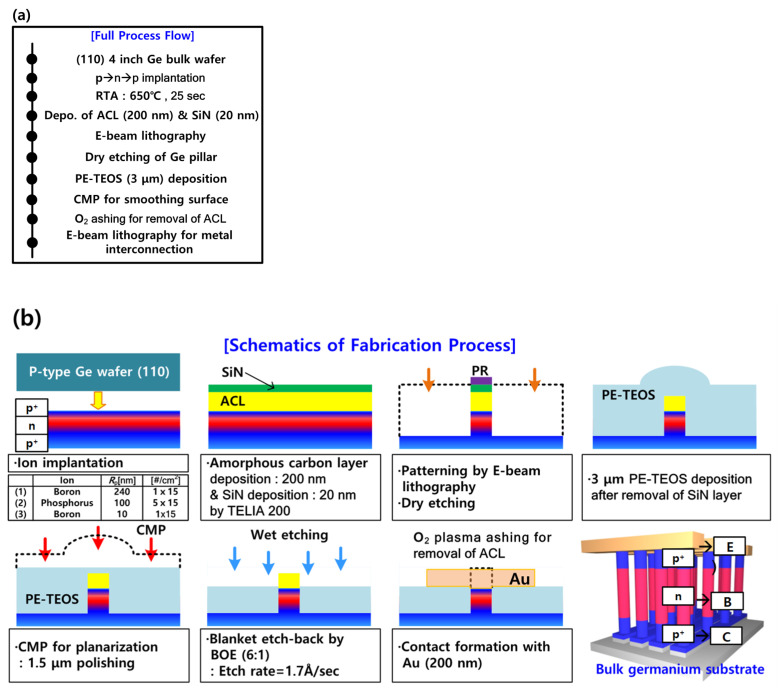
(**a**) Process flow of the fabricated vertical Ge biristor with the p^+^-n-p^+^ structure by use of CMOS process technology. (**b**) Schematics of fabrication process for the vertical-type Ge biristor with open-base (floating) structure.

The process flow and relevant schematics of the vertical-type Ge biristor are shown in Figure 1. Figure 1a summarizes the overall fabrication process of the vertical Ge biristor. As shown in Figure 1b, a p-type (110) Ge bulk wafer was used as a starting material. First, boron was implanted with an energy of 80 keV and a dose of 1 × 10^15^ cm^−2^ to form the emitter (E) at the bottom of the Ge pillar. Afterwards, phosphorus was implanted with an energy of 80 keV and a dose of 5 × 10^15^ cm^−2^ to define the base (B) at the middle of the Ge pillar. Lastly, boron was again implanted with an energy of 5 keV and a dose of 5 × 10^15^ cm^−2^ to make the collector (C) at the top of the Ge pillar. Rapid thermal annealing (RTA) at 650 °C for 20 sec was conducted to activate the dopants. For a wider sensing window and a longer retention time, it is of importance to minimize the bulk defects [17]. Then, an amorphous carbon layer (ACL) of 200 nm and silicon nitride (SiN) of 20 nm were sequentially deposited to protect the Ge pillar from physical damage by the subsequent chemical mechanical polishing (CMP) process, and from chemical damage by the recess process of the following interlayer dielectric (ILD) for the blanket etch-back, performed with the aid of buffered oxide etchant (BOE). Then the Ge pillar was vertically patterned by e-beam lithography and a dry-etching process. Tetraethyl-orthosilicate (TEOS) of 3 μm was deposited by plasma-enhanced chemical vapor deposition (PECVD). The protruded PE-TEOS layer on the vertical Ge pillar was planarized by the CMP process. Next, the PE-TEOS was recessed by the BOE (6:1) until the ACL was revealed. Afterwards, the sacrificial ACL was eliminated by O_2_ plasma ashing until the top of the Ge pillar was exposed. Finally, a landing pad of Au with an area of 1 μm^2^ was patterned on both a top of the Ge pillar and Ge substrate for electrical probing by in situ scanning electron microscopy (SEM).

**Figure 2:**
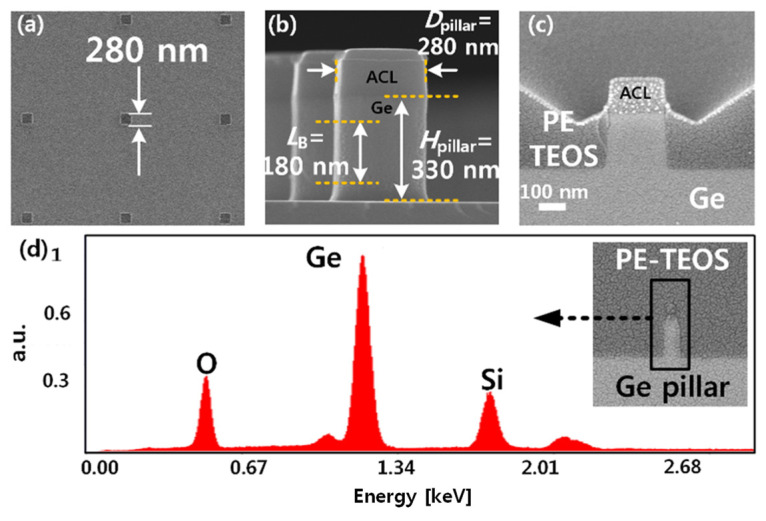
SEM images of the as-fabricated vertical Ge biristor and analysis data of the elementary components. (**a**) Top view SEM photograph. (**b**) Cross-sectional view SEM photograph. Diameter (*D_pillar_*), height (*H_pillar_*), and open-base length (*L*_B_) of the Ge pillar are 280 nm, 330 nm, and 180 nm, respectively. (**c**) Ge pillar after blanket etch-back of the PE-TEOS. (**d**) Energy dispersive spectroscopy (EDS) of the Ge pillar and PE-TEOS layer. The inset shows an SEM image of the deposited PE-TEOS on the Ge pillar.

Figure 2 presents SEM images at each fabrication step. As shown in Figure 2a,b, the Ge pillars were patterned via e-beam lithography and dry etching. The cross-sectional profile of the PE-TEOS after the blanket etch-back by BOE is shown in Figure 2c. Figure 2d shows the energy dispersive spectroscopy (EDS) data to confirm each component (Ge, Si, O). The inset of Figure 2d is a SEM image after deposition of the PE-TEOS on the vertical Ge pillar.

## 3. Experimental Results and Discussion

**Figure 3:**
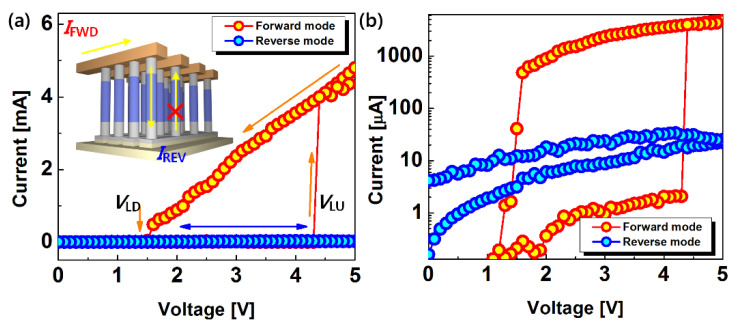
Hysteresis of the *I-V* characteristics ((**a**) linear scale, (**b**) semi-log scale) of the fabricated vertical Ge biristor for different reading directions (forward mode: collector side and reverse mode: emitter side). The bi-stable state is only observed for the forward mode. The counterclockwise loop has a *V_LU_* of 4.3 V and *V_LD_* of 1.5 V.

**Figure 4:**
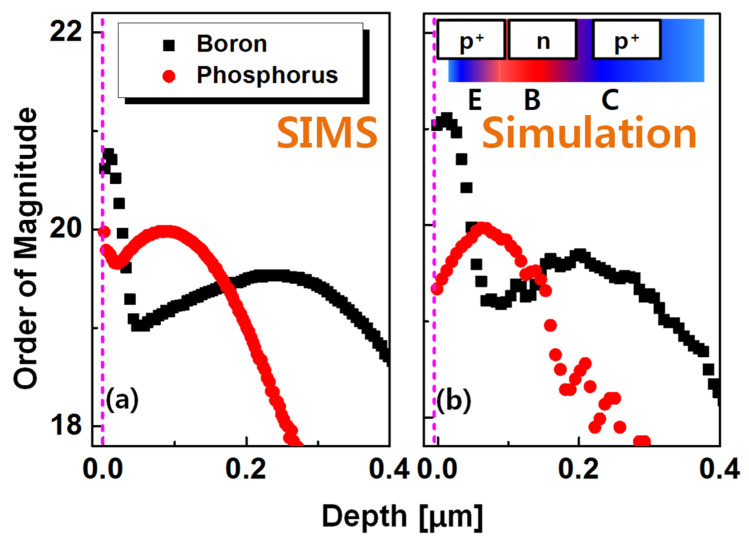
The asymmetric doping profile of the vertical Ge biristor from (**a**) measured SIMS data and (**b**) SILVACO simulation data.

Figure 3 shows the hysteric current-voltage (*I-V*) curves of the fabricated vertical Ge biristor. The distinctive and stable binary state arises from the abrupt increase in current produced by the impact ionization. The generated electrons lower the channel barrier, allowing more channel carriers to cross, leading to a positive-feedback process [7]. This abrupt current (*I*_ON_) change occurs at the latch-up voltage (*V*_LU_). Then, the generated minority carriers (electrons in our device) disappear by the recombination process and the diffusion into junction, which determines *V*_LD_. In the same bias condition, the latch-up process only occurs in a forward mode via the impact ionization. In the reverse mode, as shown in the inset of the Figure 3, the latch process is inhibited because of the asymmetric doping profile (Figure 4). It is noteworthy that the above-mentioned unidirectional property of the proposed two-terminal memory cell blocks off reverse current (*I*_REV_) through the sneaky path among neighboring cells in the cross-bar array, which enables low-power operation. This feature allows realization of a 4F^2^ memory architecture because the proposed vertical biristor with the asymmetric doping does not require an external switching element, such as a transistor or a diode.

Figure 4 shows the asymmetric doping profile of the fabricated vertical Ge biristor. Figure 4a shows the secondary ion mass spectroscopy (SIMS) data, providing a junction profile along the vertical Ge pillar. As aforementioned, this inherently non-uniform and asymmetric doping profile along the vertical direction of the Ge pillar allows the unidirectional operation of the two-terminal BJT in a cross-bar array, resulting in suppression of *I*_REV_ via the sneaky paths. This asymmetric doping profile was also verified by SILVACO simulation, as shown in Figure 4b. It can be seen that the actual doping profile obtained from SIMS and the profile obtained from simulation generally match well. High dose of ion implantation adversely induced defects thus dopant diffusion led by thermal post-annealing is affected by the process-induced defects. It is speculated that a difference between the SIMS and the simulation data is attributed to the defects. Since the doping concentration near the upper side of the pillar is higher than that near the bottom side of the pillar, the common-emitter gain (*β*) and the multiplication factor (*M*) of the forward read (FWD) are higher than those of the reverse read (REV) [8]. The latch-up action of the biristor is based on a positive-feedback process, which originates from the iterative impact ionization. The positive-feedback process can be activated by the magnitude of the electron current generated in the base region (*I*_B_) and in the collector region (*I*_C_) [7,8].

**Figure 5:**
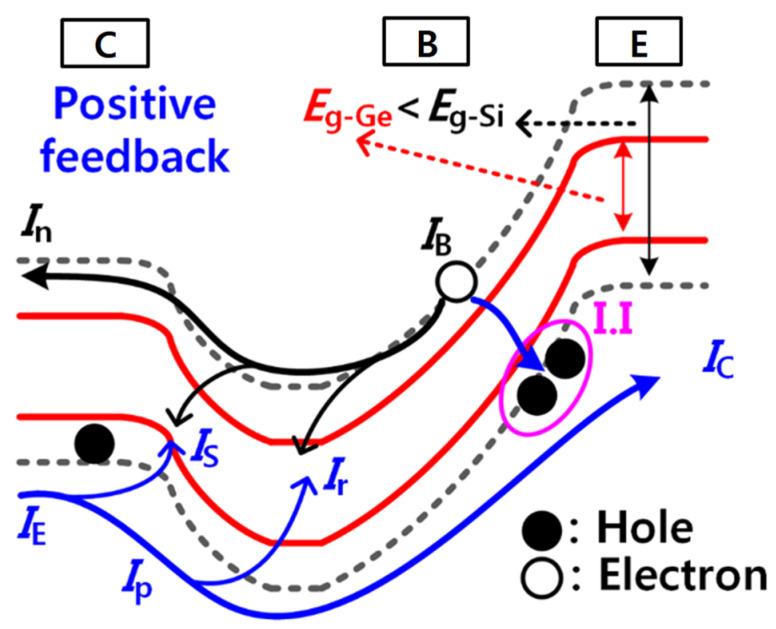
The energy band diagrams along the depth direction of the vertical structure with the different bandgap energies of Ge (red solid line) and Si (gray dashed line). *I*_n_ is the electron current injected from the base into the emitter, *I*_p_ is the hole current injected from the emitter into the base, *I*_r_ is the base recombination current, and *I*_S_ is another recombination current within the forward-biased emitter-base depleted region.

Figure 5 shows an energy band diagram along the vertical direction of the proposed Ge biristor. As shown in Figure 5, holes from the collector generate electron-hole (*e-h*) pairs through I.I near the emitter. The generated holes are flown to the emitter and the created electrons are remained in the base. Thus, the electric potential of the base is lowered. With this reduced potential, more holes are injected from the collector, the I.I rate is further increased again. As a consequence, positive feedback that can lead a latch-up phenomenon is enabled. Due to the positive feedback, current is abruptly increased at *V*_LU_, which allows binary memory operation. Because the common-emitter gain (*β*) is affected by the increased I.I rate due to the lower bandgap energy of Ge (*E*_g−Ge_ ≈ 0.67 eV) compared with that of Si (*E*_g−Si_ ≈ 1.12 eV) [13], the Ge-based biristor can operate with lower voltage and higher current.

**Figure 6:**
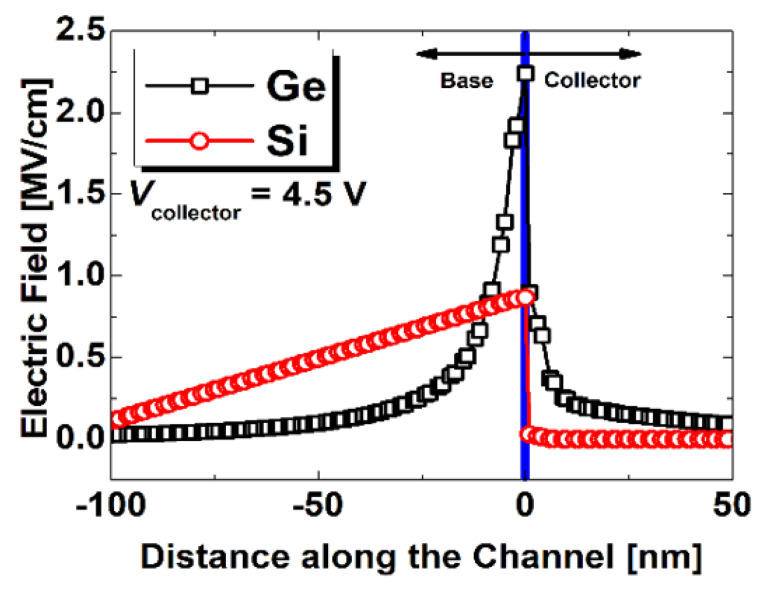
2-D SILVACO device simulation data showing e-field near the collector region of the Si and Ge biristor.

2-D SILVACO device simulation data showing electric field (e-field) near the collector region of the Si and Ge biristor is shown in Figure 6. When the same bias is applied (*V*_C_ = 4.5 V), Ge has a higher e-field than Si. Thus, it is more useful for positive feedback mechanisms and leads to lower *V*_LU_.

**Figure 7:**
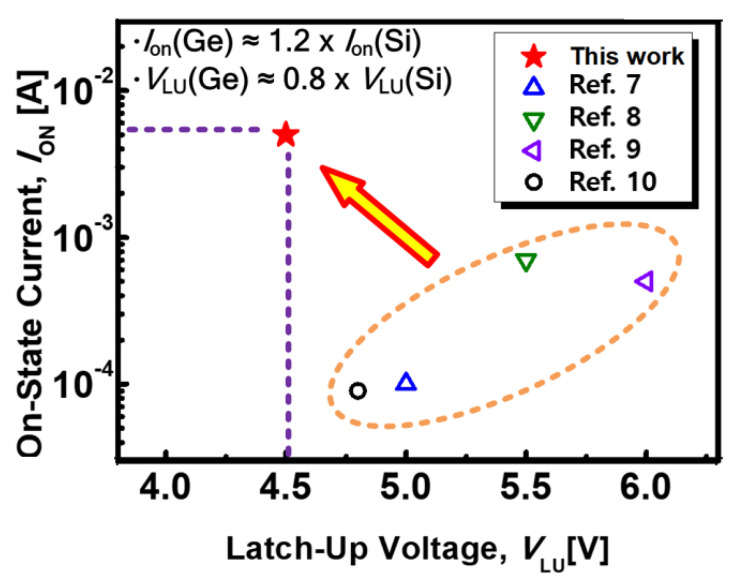
Measured *I*_ON_ and the *V*_LU_ compared with the reported I-V characteristics from the Si biristor. The filled symbol indicates the Ge-based vertical biristor in this work, while the blank symbol represents reported Si-based planar and vertical biristors.

Finally, the measured *I*_ON_ and the *V*_LU_ from the Ge biristor are compared with those of a previously reported Si biristor, as shown in Figure 7. Because of the pure Ge, the narrow bandgap of the vertical Ge biristor shows both a low *V_LU_* and a high *I_ON_*. It is worth investigating biristor characteristics of Ge for various crystal orientations, as a further work.

## 4. Conclusions

A vertical Ge biristor with a gateless p-n-p structure (base open pnp BJT), which can be applied in the gateless and capacitorless DRAM, was demonstrated for the first time. By adopting a vertical structure and pure Ge material, this memory device has the advantages of an inherently small cell size (<4F^2^) and low-voltage operation. Due to the asymmetric doping profile inside the vertical pillar, bi-stable operation is only observed for the forward mode. Thus, such unidirectional characteristic cuts off a sneaky path among the neighbored devices. Furthermore, the proposed Ge biristor showed higher *I*_ON_ and lower *V*_LU_ compared to the Si biristor. This proposed Ge biristor-based memory architecture can be used for various applications including embedded and stand-alone memory, and provide extremely long endurance, due to the intrinsic gateless structure, without a gate and gate dielectric. Therefore, the proposed Ge biristor can provide a guide as a next-generation memory device.
